# Social Determinants of Health among Older Korean Immigrants in the United States: A Systematic Review

**DOI:** 10.1093/geroni/igab046.3436

**Published:** 2021-12-17

**Authors:** Sojeong Lee, Victoria Rizzo

**Affiliations:** 1 University at Albany, State University of New York, Albany, New York, United States; 2 University at Albany, SUNY, Albany, New York, United States

## Abstract

The visible impact of the SDoHs on health and behavioral health as well as health disparities among minority populations is heightened due to COVID-19. One group about which little is known in relation to SDoHs is the older Korean immigrant population in the U.S. To examine the impact of SDoHs on the health, mental health, and health care utilization, a systematic review of studies focused on SDoHs for this population was conducted. Using multiple indexing terms, databases were searched for articles published in English between January 1, 2011 and December 2020. Articles were included in the search if they examined social determinants of health of older Korean immigrants defined as foreign-born Koreans aged 60 or older who live in the United States regardless of citizenship or legal immigration status. A total of 1090 articles were identified in the search. A review of abstracts for inclusion criteria resulted in 118 articles for review. Seventy-one articles were excluded during the review process. A total of 47 articles met inclusion criteria and were evaluated. The review revealed that SDoHs, including education level, financial resources, access to health insurance, level of acculturation and level of social support, influenced cognitive status, depressive symptoms, health status and quality of life. These findings validate the need for interventions to address the social care needs of older Korean immigrants and can be used to identify the role of social workers in addressing the SDoHs that result in health disparities for older Korean immigrants.

